# Metabolic profiling and biological activities of the aerial parts of *Micromeria imbricata* Forssk. growing in Saudi Arabia

**DOI:** 10.1016/j.sjbs.2021.05.077

**Published:** 2021-06-04

**Authors:** Hanan M. Al-Yousef, Omer I. Fantoukh, May A. El-Sayed, Musarat Amina, Rasha Adel, Wafaa H.B. Hassan, Sahar Abdelaziz

**Affiliations:** a Department of Pharmacognosy, College of Pharmacy, King Saud University, P.O. Box 2457, Riyadh, 11451 Saudi Arabia; b Department of Pharmacognosy, Faculty of Pharmacy, Zagazig University, 44519 Zagazig, Egypt

**Keywords:** *Micromeria imbricata*, Lamiaceae, Phytochemicals, Antioxidant, Cytotoxicity, Anti-obesity, UPLC-ESI-MS/MS

## Abstract

The hydroalcoholic extract (MIT) of *Micromeria imbricata* (Forssk.) growing in Saudi Arabia in addition to the chloroform (MIC) and *n*-butanol (MIB) fractions were investigated for the first time using UPLC-ESI-MS/MS. The analysis revealed the tentative identification of fifty-eight compounds including three organic acids, twenty-five phenolic compounds, three coumarins, two anthocyanins, twenty-one flavonoids, three terpenes, and one miscellaneous. Moreover, the therapeutic potential of *M. imbricata* (MIT) and its fractions (MIC and MIB) were determined by *in vitro* evaluation of their cytotoxic, antioxidant, and anti-obesity characteristics. The MIT extract showed the highest phenolic (125.23 ± 0.87 mg gallic acid equivalent/100 gm extract) and flavonoid (112.24 ± 2.45 mg quercetin equivalent/100 gm extract) contents followed by *n*-butanol and chloroform fractions. The MIT extract revealed a potent cytotoxic activity against HepG-2 (Hepatocellular carcinoma) and MCF-7 (Breast carcinoma) with IC_50_ 28.5 ± 2.0 and 35.2 ± 1.2 µg*/*mL, respectively. Additionally, the tested hydroalcoholic extract exhibited a significant DPPH scavenging activity with SC_50_ 28.4 ± 1.2 µg/mL and a remarkable lipase inhibitory activity with IC_50_ 54.2 ± 1.2 µg/mL. In conclusion, the current study presents the first insights into the phytochemical constituents and pharmacological properties of *M. imbricata* extract and its chloroform and *n*-butanol fractions. The results revealed that *M. imbricata* hydroalcoholic extract might be a prolific source of bioactive constituents with potent antioxidant, cytotoxic and anti-obesity potential. It might be a natural alternative therapy and nutritional strategy for obesity treatment.

## 1 Introduction

Genus *Micromeria* Benth. comprises 130 species belonging to Lamiaceae (mint) family. Plants of this genus are perennial herbs, shrubs and subshrubs (Brahmi et al., 2017). In traditional folk medicines, *Micromeria* species are used in different ailments like skin infections, wounds, colds, headache, fever, asthma, heart, respiratory and digestive systems disorders. They also have many biological actions including anti-inflammatory, antimicrobial, antifungal, antiseptic, antispasmodic, antioxidant and antitumor activities (Brahmi et al., 2017, Vladimir-Knežević et al., 2011, Azab, 2016). Essential oils, flavonoids and triterpenes are the main phytochemical constituents reported from the genus *Micromeria* (Vladimir-Knežević et al., 2011, Slavkovska et al., 2005). Most of the studies on plants of this genus reported their volatile constituents and their antimicrobial properties (Marinković et al., 2002, Stojanović et al., 2006, Šavikin et al., 2010). Genus *Micromeria* is widespread in South Africa, West Asia, the Mediterranean region and the Canary Islands. Moreover, there is also a high similarity between the plants of this genus. *M. imbricata* is similar to *M. fruticosa* and *M. graeca* but they have elliptic not ovate leaves, longer calyces, and narrower calyx lobes (Ryding, 2007). To date, there is no report about the phytoconstituents and the biological activities of *M. imbricata* growing in Saudi Arabia. Therefore, the chemical composition of *M. imbricata* hydroalcoholic extract as well as the chloroform and *n*-butanol fractions were analyzed for the first time using UPLC-ESI-MS/MS to identify its chemical constituents. Additionally, the biological activities including the antioxidant, cytotoxic and anti-obesity were investigated.

## 2 Materials and methods

### 2.1 Plant material

The aerial parts of *M. imbricata* (Forssk.) were collected from Abha, fertile Asir mountains of South-Western Saudi Arabia (18^o^13ʹ1″N 42^o^30ʹ19″ E), in August 2008. Plant material was identified by Dr. Mohamed Yousef, professor of Pharmacognosy from the Pharmacognosy Department, College of Pharmacy of King Saud University, and a voucher specimen was deposited in the herbarium of the same department under registry number (MI-2543).

### 2.2 Preparation of *M. Imbricata* crude hydroalcoholic extract

Ethanol 95% was used for the extraction of the air-dried powdered aerial parts of *M. imbricata* (1.5 kg). The dried hydroalcoholic extract was defatted with petroleum ether (4 × 1 L). The aqueous defatted hydroalcoholic extract (120 g) was fractionated using chloroform (CHCl_3_) and *n*-butanol (*n*-BuOH), to give 30.1 g and 32.5 g of chloroform and *n*-butanol fractions respectively.

### 2.3 Quantitative measurement of the total phenolic and flavonoid contents of *M. imbricata* extract/ fractions

Total phenolic content was determined by the Folin-Ciocalteau method (Chen et al., 2014) and expressed in milligram gallic acid equivalents per gram of dried extract/fractions (mg GAE/g extract/fractions GAE). Total flavonoid content was determined using the aluminum chloride-potassium acetate colorimetric assay with quercetin as standard (Hossain and Rahman, 2011), and the total flavonoid content was expressed as mg of quercetin equivalents per gram of extract/fractions (mg QE/g extract or fractions).

### 2.4 UPLC- ESI- MS/MS instrument and separation technique

The hydroalcoholic extract of *M. imbricata* and its chloroform and *n*-butanol fractions were prepared as a solution of 100 μg/mL using HPLC grade methanol, filtered using a membrane disc filter (0.2 μm) then subjected to LC-ESI-MS analysis in negative and positive ion acquisition modes using a (XEVO TQD triple quadruple instrument) mass spectrometer. The UPLC system was a Waters Corporation, Milford, MA01757 U.S.A. The reversed-phase separations were performed on a (ACQUITY UPLC - BEH C 18 1.7 µm - 2.1 × 50 mm Column. (50 mm x 1.2 mm [inner diameter] and 1.7 µm particle size) and at 0.2 mL\min flow rate. The used analysis parameters and the gradient program were previously reported by (Hassan et al., 2019). The identification of the phytochemical constituents was done by their fragmentation patterns and their ESI- QqQLIT–MS/MS spectra. Peaks and spectra were processed using the Maslynx 4.1 software and tentatively identified by comparing their retention time (*R_t_*), mass spectrum with the published data and Library search, such as ChemSpider (http://www.Chemspider.com), MassBank (http://www.massbank.eu), METLIN (https://metlin.scripps.edu/) and FooDB (http://www.Foodb.ca).

### 2.5 Antioxidant assay

The antioxidant activity of *M. imbricata* hydroalcoholic extract and its fractions was determined at the Regional Center for Mycology and Biotechnology (RCMB) at Al- Azhar University using the free radical 2,2-diphenyl-picrylhydrazyl (DPPH) scavenging assay (Al Khateeb et al., 2017).

### 2.6 Cytotoxicity assay

The cytotoxic effects of the hydroalcoholic extract of *M. imbricata* in addition to its chloroform and *n*-butanol fractions against HepG-2 and MCF-7 cells were carried out using the MTT cell viability assay (Ramos-Silva et al., 2017). HepG-2 (Human hepatocarcinoma) and MCF-7 (human breast carcinoma) cells were obtained from VACSERA Tissue Culture Unit and maintained in DMEM supplemented with 10% FBS and 100 μg/mL penicillin–streptomycin-amphotericin B solutions.

### 2.7 In vitro anti-obesity activity using pancreatic lipase inhibitory assay

The lipase inhibition activity of plant extract was determined as the method proposed by (Kim et al., 2010)**.** Briefly, the porcine pancreatic lipase activity was measured using *p*-nitrophenyl butyrate (NPB) as a substrate. Lipase solution (100 µg/mL) was prepared in a 0.1 mM potassium phosphate buffer (pH 6.0). To determine the lipase inhibitory activity, samples with different concentrations (1000 to 7.81 μg/mL) were preincubated with 100 µg/mL of lipase for 10 min at 37 °C. The reaction was then started by adding 0.1 mL NPB substrate after incubation at 37 °C for 15. The amount of *p*-nitrophenol released in the reaction was measured using Multiplate Reader. Each experiment was performed in triplicates. The results were expressed as percentage inhibition, which was calculated using the formula; Inhibitory activity (%) = (1-As/Ac) × 100, where is the absorbance in the presence of test substance and Ac is the absorbance of control. The IC_50_ value is defined as the concentration of *α*-glucosidase inhibitor to inhibit 50% of its activity under the assay conditions.

## 3 Results

### 3.1 Total phenolic and flavonoid contents

As shown in Table 1, the hydroalcoholic extract and *n*-butanol fraction of *M. imbricata* possessed the highest concentration of phenolic and flavonoid contents (125.23 ± 0.87 and 112.24 ± 2.45 (mg GAE/g extract) and 89.25 ± 1.75 and 81.15 ± 0.08 (mg QE/g extract) respectively. On the other hand, the chloroform fraction had the lowest concentration 20.43 ± 0.89 (mg GAE/g extract) and 6.35 ± 0.52 (mg QE/g extract).

**Table 1:** Total phenolic and flavonoid contents of *M. imbricata* hydroalcoholic extract (MIT), chloroform (MIC) and *n*-butanol (MIB) fractions.

**Extract/Fraction**	**Total phenols (mg GAE/g of ext.)**	**Total flavonoids (mg QE/ g of ext.)**
MIT	125.23 ± 0.87	112.24 ± 2.45
MIC	20.43 ± 0.89	6.35 ± 0.52
MIB	89.25 ± 1.75	81.15 ± 0.08

**GAE** gallic acid equivalent**, QE** quercetin equivalent.

### 3.2 Tentative identification of polyphenols and other constituents by UPLC-ESI-MS/MS

In this study, crude hydroalcoholic extract, chloroform and *n*-butanol fractions of *M. imbricata* were analyzed by UPLC-ESI-MS/MS, operating in both positive and negative ionization modes. The identification of abundant compounds of these extracts was based on mass fragmentation patterns and the standards data reported in the literature and database. Fifty-eight compounds were tentatively identified in the three samples of *M. imbricata* including three organic acids, twenty-five phenolic compounds, three coumarins, two anthocyanins, twenty-one flavonoids, three terpenes, and one miscellaneous. Table 2 indicates all the identified compounds, their retention times, experimental *m*/*z* in positive and negative ionization mode and MS/MS fragments.

**Table 2:** Metabolites identified in *M. imbricata* total hydroalcoholic extract (MIT), chloroform (MIC) and *n*-butanol (MIB) fractions using UPLC-ESI-MS/MS analysis in positive and negative ionization modes.

**No.**	**R_t_ (min)**	**Compound name**	**[M−H]^-^(*m*/*z*)**	**[M + H]^+^ (*m*/*z*)**	**MS/MS (*m*/*z*)**	**MIT**	**MIC**	**MIB**	**Ref.**
1	0.94	Umbelliferone		163	135, 117	+			**(**Abu-Reidah et al., 2019, Tine et al., 2017**)**
2	1.05	Eupatorin or Eupatilin		285	268, 165, 117	+			**(**Abu-Reidah et al. 2019**)**
3	1.40	Apigenin-7-*O-*glucuronide	445		269	+		+	**(**Abu-Reidah et al. 2019**)**
4	2.22	Syringic acid	197		179, 151, 135	+		+	**(**Abu-Reidah et al. 2019**)**
5	6.57	Syringic acid isomer	197		179, 135,123	+			**(**Abu-Reidah et al. 2019**)**
6	6.76	*p*-Hydroxy benzoic acid		139	121, 111, 105, 97, 79	+			**(**FooDB 2020**)**
7	7.32	Syringic acid hexoside	359		197	+		+	**(**Abu-Reidah et al. 2019**)**
8	7.49	Rosmaric acid	359		197, 179, 161	+			**(**Abu-Reidah et al. 2019**)**
9	7.59	Kaempferol or luteolin-*O*-rutinoside		595	287	+		+	**(**Abu-Reidah et al. 2019**)**
10	7.62	Malvidin derivative		621	331	+			**(**Stein-Chisholm et al. 2017**)**
11	7.85	Syringic acid derivative	377		197	+		+	**(**Abu-Reidah et al. 2019**)**
12	8.11	Umbelliferone isomer		163	135, 117	+			**(**Abu-Reidah et al., 2019, Tine et al., 2017**)**
13	8.24	Peonidin-3-(*p-*coumaroyl-glucoside)		609	301	+			**(**Stein-Chisholm et al. 2017**)**
14	8.56	Loliolide		197	179, 161, 135, 107	+	+		**(**Abu-Reidah et al. 2019**)**
15	8.82	Cirsilineol		345	329, 315	+			**(**Abu-Reidah et al. 2019**)**
16	9.20	Succinic acid	117		99, 73	+		+	**(**Al Kadhi et al. 2017**)**
17	9.36	Kaempferol-3-*O*-glucuronide	461		285	+		+	**(**Davis et al. 2006**)**
18	9.39	Isorhamnetin-*O*-rutinoside	623		477, 315	+			**(**Abu-Reidah et al. 2019**)**
19	10.35	Apigenin-7-*O*-glucuronide isomer	445		269	+		+	**(**Abu-Reidah et al. 2019**)**
20	10.49	Umbelliferone isomer		163	135, 117, 107, 89	+			**(**Abu-Reidah et al., 2019, Tine et al., 2017**)**
21	10.76	Syringic acid hexoside isomer	359		197	+	+		**(**Abu-Reidah et al. 2019**)**
22	10.80	Rosmaric acid isomer	359		197, 179, 161	+		+	**(**Abu-Reidah et al. 2019**)**
23	10.81	Kaempferol or luteolin rutinoside isomer		595	287	+			**(**Abu-Reidah et al. 2019**)**
24	10.81	Acacetin-7-*O-*rutinoside (Linarin)		593	285	+			**(**Abu-Reidah et al. 2019**)**
25	10.86	Syringic acid isomer	197		179, 135, 123	+			**(**Abu-Reidah et al. 2019**)**
26	11.15	Salvianolic acid E	717		537,519,339, 321,313,295, 197, 179	+			**(**Lopes et al., 2018, Yang et al., 2015**)**
27	11.55	Tyrosol		121	103, 93, 89, 79, 77, 73	+	+		**(**Lambert et al. 2015**)**
28	11.91	Caffeic acid	179		179, 135	+	+		**(**Quifer-Rada et al. 2015**)**
29	12.19	Apigenin		271	153	+			**(**Abu-Reidah et al. 2019**)**
30	12.26	Caffeoylquinic acid		355	163, 135	+			**(**Abu-Reidah et al. 2019**)**
31	12.53	Salvianolic acid A	493		313, 295, 135	+		+	**(**Pereira et al., 2018, Yang et al., 2015**)**
32	12.65	Acacetin-7-*O*-rutinoside isomer (Linarin)		593	285	+		+	**(**Abu-Reidah et al. 2019**)**
33	13.11	Dihydroxy-trimethoxyflavone		345	330, 315, 284	+			**(**Abu-Reidah et al. 2019**)**
34	13.20	Salvianolic acid B	717		537, 519, 339, 321, 313, 295, 197	+			**(**Yang et al. 2015**)**
35	14.04	Apigenin isomer		271	153	+	+		**(**Abu-Reidah et al. 2019**)**
36	14.58	Tyrosol isomer		121	93, 89, 79, 77	+	+		**(**Lambert et al. 2015**)**
37	14.60	Dihydroxy-trimethoxyflavone isomer	343		328, 313	+	+		**(**Abu-Reidah et al. 2019**)**
38	14.78	Rosmaric acid isomer	359		197, 179, 161	+		+	**(**Abu-Reidah et al. 2019**)**
39	14.81	Acacetin		285	285, 242	+			**(**Abu-Reidah et al., 2019, Kim et al., 2016**)**
40	15.16	Caffeic acid isomer	179		179, 135	+			**(**Quifer-Rada et al. 2015**)**
41	16.46	Acacetin isomer		285	285, 242	+	+		**(**Abu-Reidah et al., 2019, Kim et al., 2016**)**
42	16.82	Acacetin-7-*O*-rutinoside (Linarin) isomer		593	285	+			**(**Abu-Reidah et al. 2019**)**
43	17.12	Acacetin-7-*O*-rutinoside (Linarin) isomer		593	285	+		+	**(**Abu-Reidah et al. 2019**)**
44	17.17	Syringic acid isomer	197		179, 151, 135	+		+	**(**Abu-Reidah et al. 2019**)**
45	19.46	Corosolic acid	471		427	+	+		**(**Abu-Reidah et al. 2019**)**
46	19.77	Succinic acid isomer	117		100, 99		+		**(**Al Kadhi et al. 2017**)**
47	19.83	*p*-hydroxybenzoic acid isomer		139	121, 111, 97, 93	+			**(**FooDB 2020**)**
48	20.42	Apigenin isomer	269		269, 225, 149	+	+		**(**Plazonić et al. 2009**)**
49	21.15	Corosolic acid isomer	471		427	+	+		**(**Abu-Reidah et al. 2019**)**
50	21.52	Tyrosol isomer		121	93, 77, 73, 45	+			**(**Lambert et al. 2015**)**
51	23.63	Syringic acid isomer	197		179, 151, 135	+		+	**(**Abu-Reidah et al. 2019**)**
52	23.67	Tyrosol isomer		121	93, 79, 77	+	+		**(**Lambert et al. 2015**)**
53	24.85	8-Prenylnaringenin derivative	517		339	+	+		**(**Quifer-Rada et al. 2015**)**
54	25.19	Tuliposide B		295	591 (2 M + H)^+^, 143	+	+		**(**Abu-Reidah et al. 2019**)**
55	25.81	Succinic acid isomer	117		99, 73	+	+	+	**(**Al Kadhi et al. 2017**)**
56	26.50	Eriodictyol	287		151	+	+		**(**Farag et al. 2016**)**
57	26.69	Tyrosol isomer		121	93, 79, 77	+			**(**Lambert et al. 2015**)**
58	27.92	Tyrosol isomer		121	93, 79, 77	+	+		**(**Lambert et al. 2015**)**

MIT = *M. imbricata* total hydroalcoholic extract; MIC = *M. imbricata* chloroform fraction; MIB = *M. imbricata n*-butanol fraction.

### 3.3 Antioxidant activity

**Fig. 1:**
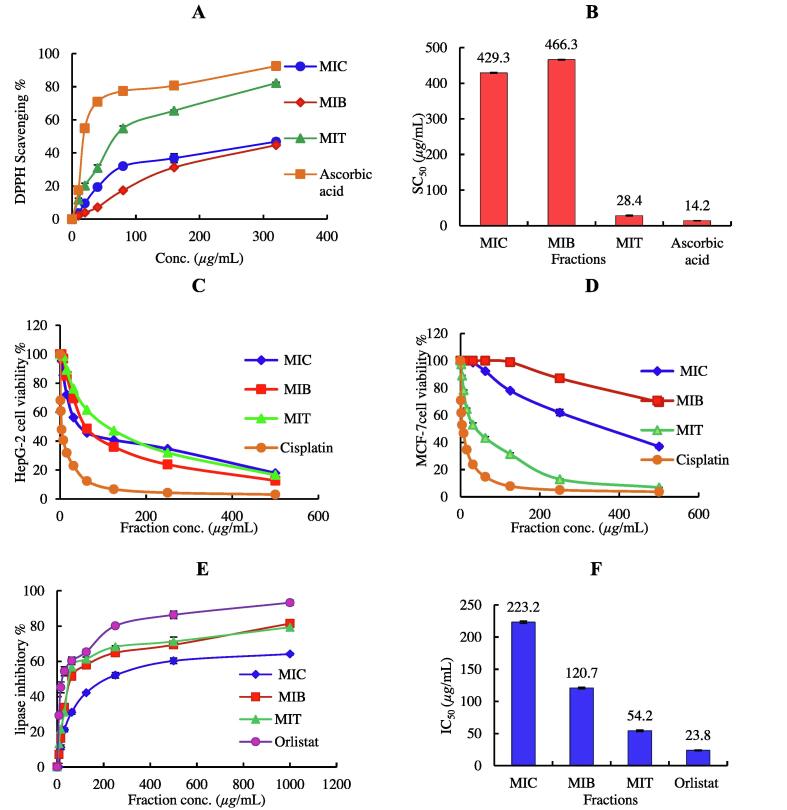
**(A):** 2,2-diphyenyl-picrylhydrazyl (DPPH) radical scavenging activity at different concentrations (10–320 µ*g*/mL) of *M. imbricata* extract and its fractions Data is presented as average ± standard deviation from three experiments. (**B):** SC_50_ of antioxidant activity *M. imbricata* extract and its fractions and ascorbic acid. **(C):** Cytotoxic activity of *M. imbricata* extract and its fractions against HepG-2 cell line at different concentrations **(D):** Cytotoxic activity of *M. imbricata* extract and its fractions against MCF-7 cell line at different concentrations. **(E):** In vitro lipase inhibitory activity of *M. imbricata* extract and its fractions compared to orlistat standard. **(F):** IC_50_ of *M. imbricata* extract and its fractions and orlistat. **MIT,** (hydroalcoholic extract), **MIB, (***n*-butanol fraction) and **MIC** (chloroform fraction).

In the present study, the antioxidant activity of the hydroalcoholic extract of *M. imbricata* (MIT) and its chloroform (MIC) and *n*-butanol (MIB) fractions were investigated, compared to ascorbic acid (a standard antioxidant) and summarized in Fig. 1 **A and B**. The MIT extract has the highest activity as indicated by its high percentage of DPPH scavenging (80%) at 320 μg/mL and low SC_50_ (concentration of sample required to scavenge 50% of DPPH radicals) 28.4 ± 1.2 µg/mL compared to MIC and MIB with SC_50_ 429.3 ± 1.3 and 466.3 ± 0.8 µg/mL, respectively, with ascorbic acid SC_50_ 14.2 ± 0.5 μg/mL as standard..

### 3.4 Cytotoxicity

The cytotoxic activity *M. imbricata* extract and its fractions against HepG-2 (hepatocellular carcinoma) and MCF-7 (breast carcinoma) cell lines using MTT assay and cisplatin as a positive standard were studied. As shown in **(**Fig. 1 **C & D)**, all the tested samples showed dose-dependent cytotoxicity against HepG-2 and MCF-7 cells. However, the MIT extract showed potent cytotoxic activity against HepG-2 and MCF-7 cells with IC_50_ 28.5 ± 2.0 and 35.2 ± 1.2 µg/mL compared to cisplatin with IC_50_ 3.67 ± 1.70 and 5.71 ± 1.30 μg/mL, respectively.

The MIC fraction had weak cytotoxicity against HepG-2 and MCF-7 cells with IC_50_ 234.0 ± 0.9 and 369.0 ± 3.2 µg/mL, respectively **(**Table 3**)**.

**Table 3:** Half maximum inhibitory concentration (IC_50_) of *M. imbricata* chloroform (MIC), *n*-butanol (MIB) fractions and hydroalcoholic extract (MIT) in cell viability of HepG-2 and MCF-7 cells after the treatment for 48 h, as measured by MTT assay. The data are presented as *µg*/mL.

**Cell line**	**Tested fractions**			
	**IC_50_ (*µg*/mL)**			
	**MIC**	**MIB**	**MIT**	**Cisplatin**
**HepG-**2 (Hepatocellular carcinoma)	234.0 ± 0.9	> 500	28.5 ± 2.0	3.67 ± 1.70
**MCF-7** (Breast carcinoma)	369.0 ± 3.2	> 500	35.2 ± 1.2	5.71 ± 1.30

These are the mean of three determinations.

### 3.5 The anti-obesity activity

The inhibition of lipase activity of the tested plant extracts is described in Fig. 1 **E & F**. The hydroalcoholic extract of *M. imbricata* exhibited higher inhibitory activity (IC_50_ 54.2 ± 1.2 µg/mL) than the chloroform and *n*-butanol fractions with IC_50_ values 223.2 ± 1.7 and 120.7 ± 1.3 µg/mL, respectively which was compared with the positive control, orlistat (IC_50_ 23.8 ± 0.7 µg/mL).

## 4 Discussion

### 4.1 Total phenolic and flavonoid contents

Most biological activities of plant extracts are associated with its phenolic and flavonoid contents. These secondary metabolites are widely distributed in several plant families. They play a crucial role in the management of various human disorders (Abbaszadeh et al., 2019). Total phenolic and flavonoid content values were observably high in MIT compared to MIB. The chloroform fraction had the lowest concentration. These results were in agreement with previous studies performed on other *Micromeira* species (Vladimir-Knežević et al., 2011, Abu-Reidah et al., 2019).

### 4.2 Tentative identification of polyphenols and other constituents by UPLC-ESI-MS/MS

Fifty-eight compounds were tentatively identified in the three samples of *M. imbricata.* These compounds comprised phenolic acids and their derivatives, flavonoids, terpenes and coumarins.

#### 4.2.1 Organic acids

Succinic acid (**16**) and its isomers (**46** and **55**) were detected in MS spectra with a deprotonated molecular ion at *m*/*z* 117. The loss of water molecule was confirmed by an intense fragment at *m*/*z* 99 (Al Kadhi et al., 2017)

#### 4.2.2 Phenolic compounds

Twenty-five phenolic acids and their derivatives were identified in the tested fractions. As previously reported, syringic acid (**4**) and its isomers (**5, 25, 44** and **51**), syringic acid hexoside (**7**) and its isomer (**21**), syringic acid derivative (**11**) (Abu-Reidah et al., 2019) and *p*-hydroxybenzoic acid (**6)** and its isomer (**47**) (FooDB, 2020) were identified.

According to Lambert et al., 2015, precursor ion of tyrosol (**27**) and its isomers (**36, 50, 52, 57** and **58**) at *m*/*z* 121 refer to [M−H_2_O + H]^+^, and they were characterized by two fragments at *m*/*z* 93 for the phenol group and *m*/*z* 77 for an aromatic ring.

The isomers **8, 22** and **38** showed the characteristic deprotonated molecular ion at *m*/*z* 359. These compounds were identified as rosmarinic acid isomers, based on their data obtained from MS^2^ fragment ions at *m*/*z* 179 corresponding to caffeic acid and *m*/*z* 197 correlated to 2-hydroxy derivative of hydrocaffeic acid (Hossain et al., 2010). Different species of *Micromeria* and other Lamiaceae species were reported to contain rosmarinic acid, which is an ester of caffeic acid (2019 Abu-Reidah et al.).

Salvianolic acid E (**26**) and B (**34**) were detected by a characteristic deprotonated molecular ion at *m*/*z* 717. The loss of danshensu and caffeic acid units were confirmed by daughter ion at *m*/*z* 519 ([M−H]^-^-198) and *m*/*z* 537 ([M−H]^-^-180), respectively (Lopes et al., 2018), while salvianolic acid A (**31**) showed a deprotonated molecular ion at *m*/*z* 493 and characteristic daughter ions at *m*/*z* 313 ([M−H]^-^-180) and *m*/*z* 295 ([M−H]^-^-198) (Pereira et al., 2018, Yang et al., 2015).

Caffoelyquinic acid (**30)** exhibited a protonated molecular ion *m*/*z* at 355 ([M + H]^+^), and a characteristic fragment ion at *m*/*z* 163 indicating the presence of caffeic acid moiety after a neutral loss of quinic acid (Abu-Reidah et al., 2019). Two isomers of caffeic acid (**28** and **40**) showed a deprotonated molecular ion at *m*/*z* 179 and MS^2^ fragment at *m*/*z* 135 ([M−H]^-^ - CO_2_), which was identical to the reported data of this compound (Quifer-Rada et al., 2015).

#### 4.2.3 Coumarin derivatives*:*

Simple coumarins (**1, 12** and **20**) were found in the hydroalcoholic extract of *M. imbricata* and identified as umbelliferone. They showed a protonated molecular ion peak at *m*/*z* 163 and MS^2^ fragments at *m*/*z* 135 and 107 as previously reported (Abu-Reidah et al., 2019, Tine et al., 2017).

#### 4.2.4 Anthocyanins:

Two anthocyanin derivatives have been detected in hydroalcoholic extract of *M. imbricata*. Compound **10** was tentatively identified as malvidin derivative as it exhibits a molecular ion at *m*/*z* 621. In the MS^2^ spectra, it showed a fragment ion at *m*/*z* 331, indicating the malvidin structure (Stein-Chisholm et al., 2017). Compound **13** was tentatively identified as peonidin derivative (peonidin-3-(*p*- coumaroylglucoside). It was characterized by a molecular ion peak at *m*/*z* 609 with a product ion at *m*/*z* 301 [M + H-162–146]^+^ (peonidin ion derived from the loss of a glucose (162 Da) and coumaroyl (146 Da) moieties) (Stein-Chisholm et al., 2017).

#### 4.2.5 Flavonoids:

A total of twenty-one flavonoids have been detected and identified in the hydroalcoholic extract, chloroform and *n*-butanol fractions of *M. imbricata.* These flavonoids have been reported from other *Micromeria* species such as *M. fruticosa* (Abu-Reidah et al., 2019).

Compounds **2**, **39** and **41** showed the same pseudomolecular ion [M + H]^+^ at *m*/*z* 285 and the presence of product ions at *m*/*z* 242 and *m*/*z* 165 for compound **2** and *m*/*z* 242 for compounds **39** and **41**. The results suggested that they are Eupatorin and acacetin isomers, respectively, as previously published (Abu-Reidah et al., 2019, Kim et al., 2016).

Cirsilineol (**15**) is a flavonoid aglycone with a pseudomolecular ion peak at *m*/*z* 345 and daughter ions at *m*/*z* 329 and *m*/*z* 315 in the ESI-MS/MS analysis (2019 Abu-Reidah et al.). Compounds **33** and **37** exhibited a protonated molecular ion at *m*/*z* 345 and a deprotonated molecular ion at *m*/*z* 343, respectively. They were tentatively assigned as dihydroxy-trimethoxyflavone as previously reported (Abu-Reidah et al., 2019).

The aglycone apigenin has been detected in both positive and negative ionization modes. Compounds **29** and **35** exhibited a protonated molecular ion at *m*/*z* 271 while compound **48** showed a deprotonated molecular ion at *m*/*z* 269, which were tentatively assigned as apigenin isomers as previously reported (Abu-Reidah et al., 2019). Eriodictyol aglycone has been suggested for compound **56**, based on the MS data ([M−H]^-^ at *m*/*z* 287) and the MS^2^ product ion at *m*/*z* 151 (Farag et al., 2016). Apigenin-7-*O*-glucuronide (**3)** and its isomer **(19**) were tentatively identified from the MS profile with [M−H]^-^ at *m*/*z* 445 and MS^2^ base peak fragment ion at *m*/*z* 269, which gave the loss of 176 Da (glucuronyl moiety). Apigenin-7-*O*-glucuronide has been previously reported in *M. pulegium* and *M. fruticosa* (Abu-Reidah et al., 2019). Kaempferol-3-*O*-glucuronide has been suggested for compound **17**, based on the MS data ([M−H]^-^ at *m*/*z* 461) and the MS^2^ product ion at *m*/*z* 285 (corresponds to kaempferol) after the removal of glucuronide moiety ([M−H−176]^-^) (Davis et al., 2006). Compounds **9** and **23** exhibited a protonated molecular ion at *m*/*z* 595 and a characteristic fragment ion at *m*/*z* 287 [M + H-rut.]^-^ attributed to protonated kaempferol or luteolin that was detected in the MS^2^ spectrum. Consequently, it was tentatively identified as kaempferol*-O*-rutinoside or luteolin-*O-*rutinoside (Abu-Reidah et al., 2019). Precursor ion of compound **18** was detected at *m*/*z* 623 ([M−H]^-^) and its diagnostic MS^2^ fragment ion at *m*/*z* 477 [M−H−146]^-^ related to neutral loss of rhamnose (-146 Da) moiety and at *m*/*z* 315 (isorhamnetin) [M−H−308]^-^ related to neutral loss of rutinose (-308 Da) moiety. Consequently, it was tentatively identified as isorhamnetin-*O*-rutinoside (Abu-Reidah et al., 2019). Additionally, the precursor ion of compounds **24**, **32**, **42** and **43** was detected at *m*/*z* 593 [M + H]^+^ and its characteristic MS^2^ fragment ion at *m*/*z* 285 [M−H−rut.]^-^ which was related to protonated acacetin. Therefore, they were identified as acacetin-7-*O*-rutinoside and its regioisomers (Abu-Reidah et al., 2019). Compound **53** exhibited a deprotonated molecular ion at *m*/*z* 517 and characteristic fragment ions at *m*/*z* 339 attributed to 8-prenylnaringenin that was detected in the MS^2^ spectrum. Accordingly, it was identified as 8-prenylnaringenin derivative (Quifer-Rada et al., 2015)

#### 4.2.6 Terpenoid derivatives*:*

A total of three terpenoid derivatives have been detected from the hydroalcoholic extract and chloroform fraction of *M. imbricata.* These terpenoid compounds have already been reported in the Lamiaceae family and other *Micromeria* species (Abu-Reidah et al., 2019) but for the first time in *M. imbricata.* A monoterpene lactone **14** was assigned as loliolide and characterized by molecular ion at *m*/*z* 197 ([M + H]^+^) and diagnostic fragment ions at *m*/*z* 179, 161, 135 and 107 (Abu-Reidah et al., 2019). Two triterpenoid isomers **45** and **49** showed [M−H]^-^ molecular ion at *m*/*z* 471 and tentatively identified as corosolic acid as previously reported (Abu-Reidah et al., 2019).

#### 4.2.7 Miscellaneous compounds

Compound **54** was suggested to be a saccharide derivative known as tuliposide B. The protonated pseudo molecular ion was observed at *m*/*z* 295 and MS^2^ ions at *m*/*z* 591 (2 M + H)^+^ and 143. Tuliposide B was previously identified in *M. fruticosa* (Abu-Reidah et al., 2019).

### 4.3 Antioxidant activity

Oxidative stress is the result of the inconsistency between free radicals and reactive metabolites production (oxidants) and their elimination by antioxidants. This inconsistency leads to the destruction of cells and organs with prospective effects on the whole organism. Antioxidants can decrease, delay or even prevent the oxidative damage through scavenging of free radicals. These reactive oxygen species and free radicals play a crucial role in the pathogenesis of different diseases such as hypertension, atherosclerosis, diabetes, cancer, and inflammatory diseases in addition to aging processes (Sharma et al., 2018, Vladimir-Knežević et al., 2011). Polyphenols are one of the most important natural antioxidants which are widely distributed in various plant families including *Lamiaceae.* This large family includes many species which are important sources of natural antioxidants (Vladimir-Knežević et al., 2011). The antioxidant activity of the hydroalcoholic extract of *M. imbricata* (MIT) and its chloroform (MIC) and *n*-butanol (MIB) fractions showed a concentration-dependent antioxidant activity as demonstrated by an increase in their DPPH radical scavenging activity. There is a proportional correlation between radical scavenging (antioxidant) activity and the total phenolic contents (Gardner et al., 2000). The MIT extract has the highest activity compared to MIC and MIB. These results are in accordance with the previous reports about the antioxidant properties of various *Micromeria* species (*Micromeria croatica, M. juliana,* and *M. thymifolia*) (Brahmi et al., 2017, Vladimir-Knežević et al., 2011). The MIT antioxidant activity could be attributed to its contents of polyphenolic compounds such as tyrosol (Al-Yousef et al., 2020, Di Benedetto et al., 2007) salvianolic acid B (Zheng et al., 2020) in addition to flavonoid contents such as eupatorin (Shin et al., 2020) and acacetin-7-*O*-rutinoside (linarin) (Xie et al., 2020). During this work, tyrosol, salvianolic acid B, eupatorin and linarin were detected in LC-MS analysis of hydroalcoholic extract.

### 4.4 Cytotoxicity

The risk of neoplasia is increasing worldwide with higher mortality rates every year. Breast cancer is the leading cause of cancer death among females. It is responsible for 15–25% of all cancer cases and deaths. Liver cancer is much more common in males. Furthermore, it is the second major cause of cancer death in men all over the world, especially in underdeveloped countries (Torre et al., 2015). Recently, plant-derived compounds possess an attractive area for many researchers attention as a promising source of many cancer lead drugs (Al-Abbasi et al., 2016, Solowey et al., 2014, Wannes et al., 2017).

*M. imbricata* extract and its fractions showed dose-dependent cytotoxicity against HepG-2 and MCF-7 cells in comparison to cisplatin. The MIT extract showed potent cytotoxic activity against HepG-2 and MCF-7 cells. This activity may be attributed to the presence of high flavonoid content as apigenin (Abbaszadeh et al., 2019), apigenin-7-*O*-glucuronide (Mazumder et al., 2020), 8-prenyl naringenin (Bailly, 2020) and linarin (Xie et al., 2020). These compounds were the predominant flavonoids showing the highest content. In addition to the high presence of corosolic acid as a triterpenoid compound (Mazumder et al., 2020). The MIC fraction had weak cytotoxicity against HepG-2 and MCF-7 cells. Their broad range effects might be a result of multiple mechanisms such as modification or interaction with several enzymes, proteins and apoptosis induction (Abdal Dayem et al., 2016, Abbaszadeh et al., 2019).

### 4.5 The anti-obesity activity

The management of obesity by natural agents is not thoroughly investigated and might be a significant substituent for producing harmless and efficient anti-obesity drugs (Nderitu et al., 2017). To the best of our knowledge, there is no report on the anti-obesity activity of *M. imbricata,* so it was deemed of interest to investigate the anti-obesity activity of the *M. imbricata* fractions using *in vitro* pancreatic lipase inhibitory assay. The hydroalcoholic extract of *M. imbricata* showed higher inhibitory activity than the chloroform and *n*-butanol fractions. This significant activity of MIT could be assigned to the existence of corosolic acid (Stohs et al., 2012), 8-prenyl naringenin (Paraiso et al., 2020), and eupatorin (Shin et al., 2020). 8-Prenyl naringenin diminishes the gain of body weight and enhances obesity-related metabolic parameters. (Paraiso et al., 2020).

Ultimately, the biological activity results indicated that the MIT extract of *M. imbricata* had the highest antioxidant, cytotoxic and lipase inhibitory activities compared to its chloroform and n-butanol fractions. This significant activity of MIT could be ascribed to the presence of polyphenolics like tyrosol, umbelliferone, salvianolic, rosmarinic, and corosolic acids in addition to the presence of apigenin glucuronide, acacetin-7-*O*-rutinoside and other flavonoids which have various therapeutic properties. It has been reported that flavonoids and other polyphenols have vital activities for human health like anticancer, antioxidant, anti-inflammatory effects in addition to their therapeutic actions in managing several obesity complications (Sekhon-Loodu and Rupasinghe, 2019, Rasheed and Azeez, 2019, Stohs et al., 2012, Di Benedetto et al., 2007).

## 5 Conclusion

The current study presents the first report about the phytochemical components and pharmacological activities of *M. imbricata* Forssk. growing in Saudi Arabia. The hydroalcoholic extract showed the highest antioxidant, cytotoxic and anti-obesity activities compared to the chloroform and *n*-butanol fractions. These activities of MIT extract could be attributed to the presence of a high percentage of flavonoids and other polyphenols. In summary, these findings revealed that the *M. imbricata* hydroalcoholic extract possessed potential antioxidant, cytotoxic and anti-obesity properties. Also, it brought to light a new lead to the limited therapeutic options of breast and liver cancers. Moreover, this work suggests that *M. imbricata* hydroalcoholic extract may be a promising candidate as anticancer, antioxidant and anti-obesity drugs. Further studies are warranted to isolate and identify the bioactive secondary metabolites from this extract using various spectroscopic and spectrometric techniques for future *in vivo* investigation.

## Authors’ Contributions

All authors made considerable contributions to the manuscript. ME, SA, WH, and RA designed the study. ME, HA, SA, MA, OF, and RA performed the experiments. ME, HA, MA, SA and WH interpreted the results. ME, HA, WH, OF, RA and SA wrote the manuscript. All authors revised the manuscript and approved it for publication.

## Declaration of Competing Interest

The authors declare that they have no known competing financial interests or personal relationships that could have appeared to influence the work reported in this paper.
